# Editorial: From causal circuit mapping to clinical translation: Prefronto‐thalamic hypoconnectivity in schizophrenia

**DOI:** 10.1002/pcn5.70423

**Published:** 2026-10-07

**Authors:** Norio Yasui‐Furukori

**Affiliations:** ^1^ Department of Psychiatry, School of Medicine Dokkyo Medical University Mibu Japan

The study by Yahata et al.,^1^ published in *Psychiatry and Clinical Neurosciences Reports* and selected for the 2025 *PCNR* Award, addresses a persistent challenge in psychiatric neuroimaging. Numerous case‐control differences in connectivity have been reported, but few are linked to a causal mechanism relevant to symptoms or treatment. Rather than starting with a human imaging association and seeking a mechanistic explanation retrospectively, Yahata et al. begin with a pathway whose function was established by selective manipulation in nonhuman primates (NHPs), then ask whether the homologous circuit is altered in people with psychiatric disorders.

That foundation was a chemogenetic study in macaques by Oyama et al.^2^ Selective silencing of projections from the dorsolateral prefrontal cortex (DLPFC) to the lateral part of the mediodorsal thalamic nucleus (MDl) impaired spatial working memory, whereas manipulation of another prefrontal‐subcortical pathway produced a dissociable effect on decision‐making. Using this circuit as an a priori target, Yahata et al.^1^ examined resting‐state functional connectivity between human DLPFC area 46 and the MDl in a harmonized multisite dataset. The analysis included 511 healthy controls, 91 patients with schizophrenia, 133 with major depressive disorder, and 120 with autism spectrum disorder. Bilateral DLPFC‐MDl connectivity was lower in schizophrenia than in healthy controls, with a modest effect size (Cohen's *d* approximately 0.3). Comparable reductions were not detected in the other two diagnostic groups or in connections between the DLPFC and several neighboring thalamic nuclei. The contribution is therefore more than another demonstration of thalamic dysconnectivity: it identifies a fine‐grained pathway selected in advance from causal experimental evidence.

Conceptually, the work fits a contemporary view of the thalamus as an active partner in cortical computation rather than a passive relay. Experimental evidence indicates that mediodorsal thalamic input can sustain prefrontal activity during working‐memory maintenance.^3^ Human imaging studies have repeatedly shown reduced connectivity of the thalamus with prefrontal and cerebellar regions, together with increased connectivity to sensorimotor cortices, across psychotic disorders.^4^ Diffusion imaging has likewise demonstrated reduced prefrontal‐thalamic anatomical connectivity in schizophrenia and linked it to working‐memory impairment.^5^ This convergent evidence supports a circuit model in which impaired coordination between associative thalamic nuclei and prefrontal cortex contributes to cognitive dysfunction. It also underscores that structural and functional connectivity reflect related, but not interchangeable, features of circuit biology.

Research is now moving from coarse regional comparisons toward pathway‐specific, stage‐sensitive, and multimodal characterization. Thalamic dysconnectivity has been associated with subsequent transition to psychosis in young people at clinical high risk,^6^ suggesting that circuit abnormalities may precede chronic illness and prolonged treatment exposure. A recent 7‐T study integrated resting‐state functional magnetic resonance imaging (MRI), magnetic resonance spectroscopic imaging, and sleep electroencephalography in individuals at clinical high risk for psychosis.^7^ Reduced mediodorsal thalamus‐DLPFC connectivity and shorter prefrontal sleep spindles jointly delineated a subgroup with poorer working memory. Such studies point beyond a single connectivity measure toward an integrated account of circuit function, neurochemistry, electrophysiology, and cognition.

Three design choices deserve emphasis. First, the NHP‐derived hypothesis narrows the search space and provides a functional basis for interpreting the human finding. Second, the multisite, multidisorder database and traveling‐subject harmonization address a major source of measurement bias in psychiatric imaging.^8^ Third, inclusion of two nonpsychotic diagnostic groups strengthens the analysis beyond a simple schizophrenia‐control comparison. Nevertheless, specificity should be interpreted within the boundaries of the sample: the result distinguishes schizophrenia from the disorders and thalamic targets examined, but does not establish that the abnormality is exclusive to schizophrenia. Bipolar psychosis, first‐episode psychosis, and clinical high‐risk states were not included, and some thalamocortical disturbances extend across the psychosis spectrum.^4^, ^6^ Causality also remains on the NHP side of the translation; the human component is observational.

The finding is thus best regarded as a mechanistic lead, not a biomarker ready for clinical use. Effect sizes were modest, and the group distributions overlapped substantially. In the schizophrenia subgroup with cognitive data (*n* = 40), DLPFC‐MDl connectivity was not significantly related to verbal memory or digit‐sequencing performance. This absence of a brain–behavior correlation tempers mechanistic interpretation, although the human tests only imperfectly matched the spatial working‐memory paradigm used in macaques, and resting‐state connectivity may not capture task‐evoked circuit recruitment. Similarly, the absence of an association with chlorpromazine‐equivalent dose does not exclude effects of cumulative exposure, illness duration, or confounding by clinical severity. A 2025 *PCNR* study by Koreki et al.^9^ likewise illustrates the value of linking a biological signal to an experimentally tractable construct. Both studies also show why mechanism must be tested at the individual level rather than inferred from group separation alone.

Translation now depends on prospective testing in independent, preregistered cohorts that span clinical high‐risk states, first‐episode psychosis, bipolar psychosis, and chronic schizophrenia. Longitudinal sampling is needed to determine whether hypoconnectivity is a vulnerability marker, a state marker, a consequence of illness progression, or a modifiable correlate of recovery. Resting‐state MRI should be complemented by spatial working‐memory tasks that more closely match the NHP paradigm, along with higher‐field imaging, individualized thalamic parcellation, diffusion imaging, spectroscopy, and sleep‐spindle measures. Open, harmonized resources such as the database used in the award‐winning study provide an important foundation,^8^ but reproducibility across scanners, countries, and clinical settings must be demonstrated. A stable multimodal signature is more likely to be clinically informative than any single resting‐state connection.

Near‐term clinical value is more likely to lie in treatment stratification and target engagement than in stand‐alone diagnosis. A circuit measure might identify patients whose cognitive impairment is accompanied by associative thalamocortical dysfunction, enrich proof‐of‐concept trials, and serve as a pharmacodynamic outcome. Scalable phenotyping will be essential. In 2026, *PCNR* reported that CAT‐Hokkaido, a brief tablet‐based battery, detected impairment in four of six tasks; all six tasks correlated with the corresponding measures of the MATRICS Consensus Cognitive Battery in patients with schizophrenia.^10^ After normative and cross‐site validation, such tools could complement imaging outcomes in circuit‐informed trials. Thalamocortical circuitry may also be modifiable: in a randomized study of cognitive remediation in chronic schizophrenia, increased thalamo‐prefrontal connectivity was associated with improvement in overall cognition.^11^ Future trials should test whether baseline DLPFC‐MDl connectivity predicts treatment response and whether connectivity change mediates durable gains in cognition and real‐world functioning.

Neuromodulation remains a longer term possibility. Stimulation of an accessible cortical node such as the DLPFC may influence the wider thalamocortical network, whereas transcranial focused ultrasound may eventually permit noninvasive modulation of individual deep nuclei. A recent human proof‐of‐concept study achieved highly focal ultrasound targeting of the lateral geniculate nucleus and demonstrated downstream effects in connected visual cortex.^12^ Those results cannot be extrapolated to schizophrenia or to the MDl, which presents distinct anatomical and functional challenges. They do, however, show that focal deep‐brain target engagement and network‐level readouts are becoming experimentally accessible in humans. Any clinical application will require sham‐controlled studies, careful safety evaluation, optimization of stimulation parameters, and outcomes centered on cognition and functioning rather than connectivity normalization alone.

The broader importance of this study lies in the translational sequence it makes visible: selective circuit perturbation in a species with homologous prefrontal organization, pathway‐specific mapping in human disease, longitudinal and multimodal validation, experimental target engagement, and ultimately demonstration of clinical benefit. Psychiatry has no shortage of correlational brain findings; it needs findings that are anatomically precise, functionally interpretable, reproducible, and actionable. Yahata et al.^1^ do not complete the journey from circuit to treatment, but they provide a credible map. That shift from cataloging dysconnectivity to testing circuits is the study's most enduring contribution and a fitting basis for the 2025 *PCNR* Award.

## AUTHOR CONTRIBUTIONS

Norio Yasui‐Furukori conceived the editorial, reviewed the literature, drafted and critically revised the manuscript, and approved the final version.

## CONFLICT OF INTEREST STATEMENT

Norio Yasui‐Furukori is the Editor‐in‐Chief of *Psychiatry and Clinical Neurosciences Reports* and the sole author of this article. To minimize bias, he was excluded from all editorial decision‐making related to the acceptance of this article for publication. N.Y.‐F. reports having received honoraria for lectures from Daiichi Sankyo, EA Pharma, Eisai, Eli Lilly and Company, Hisamitsu Pharmaceutical, Janssen Pharmaceuticals, Kowa, Lundbeck, Meiji Seika Pharma, Mitsubishi Tanabe Pharma, Mochida Pharmaceutical, MSD, Ono Pharmaceutical, Otsuka Pharmaceutical, Shionogi, Sumitomo Pharma, Takeda Pharmaceutical, Tsumura, Viatris, and Yoshitomi Yakuhin; has served in advisory or consultancy roles for EA Pharma, Bristol Myers Squibb, Boehringer Ingelheim, Janssen Pharmaceuticals, Mitsubishi Tanabe Pharma, Mochida Pharmaceutical, Sumitomo Pharma, and Otsuka Pharmaceutical; and has participated as a sub‐investigator in industry sponsored clinical trials conducted by EA Pharma and Bristol Myers Squibb.

## ETHICS APPROVAL STATEMENT

Not applicable.

## PATIENT CONSENT STATEMENT

N/A.

## CLINICAL TRIAL REGISTRATION

N/A.

## Data Availability

Data sharing is not applicable to this article because no new data were created or analyzed.
